# Engagement with advice to reduce cardiovascular risk following a health check programme: A qualitative study

**DOI:** 10.1111/hex.12991

**Published:** 2019-10-23

**Authors:** Samah Alageel, Martin C. Gulliford, Alison Wright, Bernadette Khoshaba, Caroline Burgess

**Affiliations:** ^1^ School of Population Health Sciences Faculty of Life Sciences and Medicine King’s College London London UK; ^2^ Community Health Sciences Department College of Applied Medical Sciences King Saud University Riyadh Saudi Arabia

**Keywords:** cardiovascular disease, cardiovascular prevention, NHS Health Check, qualitative research

## Abstract

**Background:**

The success of a cardiovascular health check programme depends not only on the identification of individuals at high risk of cardiovascular disease (CVD) but also on reducing CVD risk. We examined factors that might influence engagement and adherence to lifestyle change interventions and medication amongst people recently assessed at medium or high risk of CVD (>10% in the next 10 years).

**Method:**

Qualitative study using individual semi‐structured interviews. Data were analysed using the Framework method.

**Results:**

Twenty‐two participants (12 men, 10 women) were included in the study. Four broad themes are described: (a) the meaning of ‘risk’, (b) experiences with medication, (c) attempts at lifestyle change, and (d) perceived enablers to longer‐term change. The experience of having a health check was mostly positive and reassuring. Although participants may not have understood precisely what their CVD risk meant, many reported efforts to make lifestyle changes and take medications to reduce their risk. Individual’s experience with medications was influenced by family, friends and the media. Lifestyle change services and family and friends support facilitated longer‐term behaviour change.

**Conclusions:**

People generally appear to respond positively to having a CVD health check and report being motivated towards behaviour change. Some individuals at higher risk may need clearer information about the health check and the implications of being at risk of CVD. Concerns over medication use may need to be addressed in order to improve adherence. Strategies are required to facilitate engagement and promote longer‐term maintenance with lifestyle changes amongst high‐risk individuals.

## 1 INTRODUCTION

Cardiovascular disease (CVD) is a major cause of death globally^1^ and accounts for around 160 000 deaths annually in the United Kingdom (UK).^2^ A large proportion of CVD might be preventable through modification of behavioural risk factors (smoking, lack of physical activity) and taking preventive medication. There is evidence that multiple risk factor interventions for people without CVD, with or without pharmacological treatments, appear to have little impact on CVD risk and CVD risk factors^3^, ^4^ or on the risk of coronary heart disease mortality or morbidity.^5^

Evidence suggests that communicating information about disease risk to people is challenging and complex.^6^ Risk information on its own is not effective and needs to be coupled with other intervention elements to promote healthy behaviour.^7^ A systematic review of the literature of providing coronary risk information to adults suggested that whilst global risk information seems to improve the accuracy of risk perception, there is no evidence that risk perception translated into lifestyle changes.^7^ Providing risk information to health‐care providers appeared to increase medication prescribing,^7^ but the evidence is unclear of the efficacy of risk communication on promoting medication adherence.^8^ The intensity of the accompanying risk‐reducing intervention components may be important as well as whether risk information is presented repeatedly rather than only once. Adherence may be affected by an individual’s understanding of their risk and the manner in which risk‐reducing behaviour change is promoted.

A CVD risk prevention programme is being implemented across the National Health Service (NHS) in England to all adults aged 40‐74 years who do not have pre‐existing CVD, hypertension, diabetes or chronic kidney disease. Eligible adults are invited for screening consultations every five years for assessment of a range of CVD risk factors.^9^ People assessed as having a greater than 20% risk of developing CVD in the next 10 years are offered medical and behavioural interventions aimed at lowering their risk. The implicit assumption is that conveying risk information and promoting behaviour change will lead to risk‐reducing behaviours. The success of the health check programme depends not only on the identification of those at high risk of CVD but also on reducing CVD risk by engaging and maintaining high‐risk individuals in behaviour change. High‐risk individuals may be prescribed medication (eg for hypertension or hypercholesterolaemia) and/or given advice about lifestyle change, such as smoking cessation, weight management, physical activity or alcohol reduction. A systematic review of qualitative studies of patients’ experiences of the health check programme suggested that although people were satisfied with the overall programme, confusion about the purpose of the programme was reported.^10^ Patients who received the health check appeared to also be confused by the their CVD risk score, and the score seemed to have little meaning for how they perceived their health.^10^ Although participants reported receiving behaviour change interventions, many felt these were basic and needed more detail.^10^ This review has examined patients’ overall experience with the health check programme, rather than high‐risk individuals respond to risk‐reducing interventions, including lifestyle and medication uptake and adherence. Previous qualitative evidence has suggested that patients question the necessity of taking medications for the primary prevention of CVD and express concerns over medications’ side‐effects.^11^, ^12^ However, patients considered it legitimate to prescribe medications for high‐risk patients, subject to regular monitoring for side‐effects and medication effectiveness.^12^

It has been suggested that the role of risk perceptions in motivating risk‐reducing behaviour has been under‐estimated in research.^13^ However, other commentators argue that attempts to increase risk perceptions will rarely result in behaviour change.^14^ The way in which risk and other health information is presented to patients may influence adherence to medication and lifestyle change advice, and there is probably potential for improvements in delivery of information and advice to enable more informed decision making.^15^ A meta‐analysis of experimental studies suggested that the impact of risk information on behaviour change is moderated by the extent to which patients believe that behaviour change will reduce risk and their perceived ability to change.^16^ Therefore, accompanying risk information with other behaviour change techniques may result in risk‐reducing behaviour.

The health check programme provides a unique opportunity in terms of addressing multiple risk factors through risk communication, behaviour change interventions and/or medication provision that, if successful, might reduce the risk of several diseases. Although previous studies have examined patients’ experiences with the programme as a whole, focusing on higher‐risk patients’ experience is important for the programme’s outcomes. The aim of the present study was to examine higher‐risk patients’ longer‐term impressions of feedback given to them during the health check about their health, including risk factor levels, and to explore this and other factors associated with engagement in suggested risk‐reducing interventions, including medication use. We aimed to interview patients up to six months following their health check in order to identify factors related to their engagement with risk‐reducing interventions in the longer term.

## 2 METHOD

### 2.1 Study design

Qualitative study was conducted using semi‐structured interviews with people who were assessed at medium to high risk of developing CVD in the next ten years during the health check. The study was part of a wider service evaluation of UK’s NHS Health Check programme in South East London and was registered on the database of the Research Development Centre for South East London NHS Organisations at Southwark Public Health Department (RDLSL2047).

### 2.2 Study participants and sampling

Consistent with the eligibility criteria for the NHS Heath Check, eligible participants were aged between 40 and 74 years. Participants were eligible if they had received a health check in the last six months and were assessed at medium to high risk (>10% risk) of developing CVD in next 10 years and were registered with general practices across two South East London boroughs: Lewisham and Lambeth. CVD risk assessment was done using QRISK2 score.^17^ A convenience sample was employed, where potential participants were identified by general practice staff from the results of their health check and invited to take part in the study by their general practitioner (GP). Out of 353 patients who were invited, 26 agreed to participate in the study. Four patients were not recruited for logistic reasons.

### 2.3 Data collection

Topics for the interview (Supplementary material 1) were drawn from the literature on uptake and adherence to lifestyle change interventions and medication use. Items in the schedule were influenced by the Theoretical Domains Framework (TDF) which is drawn from models to explain behaviour change to understand factors that influence implementation of interventions.^18^ The TDF covers a set of domains comprising the main evidence‐based factors influencing behaviour change.^18^ As we were examining a range of behaviours, however, rather than focusing on one aspect of behaviour change, we drew also from other theories, including those concerning medication adherence (eg Clifford et al^19^) and access to health care.^20^ Interventions targeting multiple behaviours need to take account a wide variety of factors including beliefs, social influences and environmental context and resources.

Interview items were generated and agreed by members of the research team. The interview covered participants’ experience of having a health check, their understanding of their personal risk of CVD, and their feelings and attitudes about the feedback and advice given to them about lifestyle change and medication. Interviewees were asked about any changes they had made to their lifestyle, their experience of medication, where relevant, and about influences on adherence to these changes. The interview schedule remained the same throughout the interview process although the prompts and probes differed for each individual depending on their responses. Sociodemographic data were also collected at the time of interview, including age, sex, ethnic group and Index of Multiple Deprivation (IMD) rank. The English indices of Deprivation provide a measure for relative deprivation for small areas that are measured based on seven domains: income, employment, health deprivation and disability, education, housing, crime and living environment.^21^

The interviews were all undertaken by one researcher experienced in qualitative interviewing and research (CB). Participants had been sent an information sheet about the study by their GP, explaining why they had been invited. Interviews were conducted face to face at home, at the general practice, or over the telephone, according to the participant’s preference. Data collection stopped when no further participants could be recruited within the timeframe of the study.

### 2.4 Data analysis

Interviews were digitally recorded with the participant’s consent and fully transcribed. The analysis was conducted after completion of the interviews. A qualitative analysis based on the Framework method was used.^22^ The Framework method can be adapted for use with deductive or inductive analyses, and it is not aligned with a particular theoretical approach.^23^ This method adopts a ‘case by theme’ approach which involves several stages: transcription, familiarization, coding, sorting and charting the data according to important or dominant issues and themes and data interpretation.^22^ Our themes and codes were not pre‐selected in a deductive way; however, they were generated from the data using coding and refinement of themes. One member of the research team (BK) coded each transcript, giving a label to segments of the data that appeared significant or important and a random sample coded by another member of the team (CB). The two researchers met to compare coding for the first three transcripts and to agree on codes to be applied to subsequent transcripts. Categories were derived from grouping codes together to produce an analytic framework. The analytic framework was refined in an iterative way during the analysis via discussion with the research team. Finally, team meetings were conducted to ensure agreement about the themes and to assess whether the data were representative of the themes.

## 3 RESULTS

Twenty‐two participants (12 men, 10 women) were interviewed (see Table 1). Interviews were 20‐40 minutes in length.

**Table 1:** Participants characteristics

Invitation for the health check	
Invited by letter	14
Opportunistic	7
Don’t know/can’t remember	1
Sex	
Men	12
Women	10
Age	
40‐55	3
56‐70	18
Unknown	1
Smoking status	
Smoker	3
Ex‐smoker	5
Non‐smoker	14
Ethnic group	
UK White	18
African‐Caribbean	2
European	1
Mixed ethnicity	1
Index of multiple deprivation rank 2007 (quintiles)	
1: most deprived	4
2	10
3	4
4	3
5: least deprived	0
Missing	1
Interview mode	
Face to face	13
Phone	9
Borough	
Lewisham	14
Lambeth	8
Employment status	
Employed (full or part‐time)	12
Unemployed (including retired)	10
Co‐morbidities	
None recorded	13
Thyroid	1
High blood pressure	3
Mental health problems	2
Prostate cancer	1
HIV	1
Arthritis	1

Most of the participants had received a written invitation through the post for their health check, and most of these elected to have the check conducted at their general practice. Some participants were unaware that they had received a health check and had apparently received the check opportunistically whilst attending their general practice for unrelated appointment. We were unable to ascertain whether these individuals had not received explicit information about the health check or had not understood the information provided. The majority of the participants reported being offered weight management interventions, and all smokers were offered smoking cessation interventions. Two individuals reported they were already prescribed medication to reduce CVD risk at the time of their health check, so may have been invited or offered the check erroneously. They were included in the study nonetheless to assess attitudes towards taking medication and maintenance of behaviour change.

Most participants reported the experience of having a health check as positive and reassuring:

She [the nurse] was a very, very lovely lady and very informative … And wasn’t a bit, what’s the word – judgemental – about the overweight. And, you know, I was very impressed by her. Good experience, quite informal non‐judgemental (ID8, Female, aged 70, 18.8% CVD risk, 2nd IMD quintile)

For others, the process was not as they anticipated or was experienced more negatively:

So that’s what I found a bit off‐putting. I didn’t like that form filling (ID3, Female, aged 57, 19.7% CVD risk, 2nd IMD quintile)

Four broad themes were identified from the data: the meaning of ‘risk’; experiences with medication; attempts at lifestyle change; and perceived enablers to longer‐term change.

### 3.1 The meaning of ‘risk’

Whilst most study participants recalled a general discussion of risk, many could not recall specific information about this or their individual risk factors. There was evidence that whilst people did not recall their precise risk score, they did understand that their CVD risk was higher than it should be:

Well there were two nurses and they were pretty nice, they chatted a lot, we had quite a good laugh, but no I don’t remember much about it. They said my cholesterol was somewhat elevated. And my weight was a little higher than it should be.

**Did they give you any information about your risk of cardiovascular disease?**

Yes they did.

**Do you remember what that was?**

No I don’t. (ID17, Male, aged 54, CVD risk >10%, 3rd IMD quintile)

One man understood the implications of the information given regardless of recalling his risk score:

Well he basically said, I forget the exact words, ‘you’re not in the danger zone, but you’re sort of heading that way’ as it were (ID10, Male, aged 66, 10.2% CVD risk, 1st IMD quintile)

One participant contextualized her risk relative to other health conditions that she considered more serious threats.

I mean obviously being told that you’ve got high cholesterol is not like being told you’ve got a terminal disease. If you’re told you’ve got cancer and if you’re told you’ve got heart disease, it’s like not as bad as cancer is it really …? It never occurred to me that sort of thing [high BP] was dangerous but I suppose it must be. (ID7, Female, aged 65, 16% CVD risk, 1st IMD quintile)

It appeared that personal risk perceptions were related to perceived family history, and in particular, with whether individuals regarded themselves as coming from a ‘heart disease family’ or a ‘stroke family’. Perceived family history worked both ways in terms of participants’ attitudes towards their own health risk and risk‐reducing behaviour. Some pointed to longevity in their family as reason not to change their lifestyle. Others attributed their at‐risk health status to their genes and felt there was therefore nothing they could do that would alter the inevitable.

But over the years I’ve been putting on weight, and I know it’s nothing to do with my food, my diet. I believe it’s to do with a genetic problem. My dad had high cholesterol. He first experienced a heart problem when he was in his fifties. (ID3, Female, aged 57, 19.7% CVD risk, 2nd IMD quintile)

In contrast, for others perceived family history acted as a motivator for behaviour change.

When I saw Dr X … I explained about my mum and my dad and brothers, you know (having heart disease). And that’s why he was concerned. Both of my brothers, they were very sedentary. They didn’t do sports at all. And I’ve always done sports you know. They both smoke. I hope I’ve been lucky that it’s not got me… (ID2, Male, aged 69, 21% CVD risk, 4th IMD quintile)

### 3.2 Experiences with medication

Those who had been prescribed statins for high cholesterol reported a variety of side‐effects, and some were prescribed several different types of statin in an effort to overcome these. Some did not adhere to the medication or decided not to take it at the outset. Some felt it was preferable to try lifestyle changes first before ‘resorting’ to medication.

People talk a lot about statins. But it always seems to me, and I’m not a medical person, that you want to put off being on medication for as long as possible. Frankly you can do all the other avenues first because I think once you’re on it you can’t really get off again. (ID16, Male, aged 54, CVD risk >10%, unknown IMD quintile)

Some of those who were on regular medication expressed acceptance of the necessity of this as well as some concern over long‐term use. The influence of friends, family and the media was evident in some participant reports, which may contribute to ambivalence to taking statins.

I started hearing things … it was on the radio, they were talking about them, statins. And the after effects of statins and so on … there were a lot of people who were having bad after effects from the statins (ID3, Female, aged 57, 19.7% CVD risk, 2nd IMD quintile)

As well as some ambivalence about taking medication, some interviewees had first‐hand experience of adverse effects. One woman who experienced severe muscle pains persevered nonetheless accepting that taking statins would benefit her in the longer term.

My knees, it’s like having a toothache permanently in my knees. He [the doctor] said ‘you can carry on using them and put up with it or you don’t take them and you die of a heart attack or stroke’. And I’m like ‘nice’! (ID7, Female, aged 65, 16% CVD risk, 1st IMD quintile)

Others were also philosophical about the necessity of taking medication:

If it’s going to make me live longer, fine, that’s the way I look at it. I don’t want to take pills; I’ve never been a ‘pill taker’. But if its two little pills and they’re going to keep me going, fine. Then I’ll get my pension money! (ID4, Female, aged 64, CVD risk >10%, 4th IMD quintile)

Many of those adhering to regular medication reported the ease of obtaining repeat prescriptions and their medication from local pharmacies, so these practical issues did not seem to be a barrier to adherence. Having a well‐established routine for taking medication also facilitated adherence:

I’ve got it organised. Every morning I open the bedside cabinet, blood pressure pill, take that. And then of a night time, I’ve got a spare pair of reading glasses on top of my bedside cabinet and underneath them are my cholesterol pills. So I reach and there’s a rattle – oh I’ve got to take my cholesterol pill. So that’s it, my little routine. (ID4, Female, aged 64, CVD risk >10%, 4th IMD quintile)

### 3.3 Attempts at behaviour change

Regardless of their appreciation of the significance of their CVD risk, the predominant message recalled by most of those interviewed related to making changes to diet or exercise to improve health. Receiving a health check appeared to have a profound effect on some individuals who reported immediately implementing changes to their lifestyle, suggesting that the essence of the health messages had been assimilated. Some cut‐out specific foods, or started walking to work, increasing physical activity. Although this may reflect socially desirable responding rather than actual behaviour change, there were individual reports of actual weight loss or health improvements as a result of behaviour changes:

I’ve been going to Weightwatchers and I’ve lost at least a dress size … I’ve lost about, not quite a stone … And I’ve taken up walking. I got all the vouchers and everything [for free membership], because it’s quite expensive Weightwatchers, it’s not cheap, so I was happy about that. (ID21, Female, aged 69, CVD risk >10%, 3rd IMD quintile)

Obstacles to initiating and maintaining behaviour change included health issues such as eyesight and mobility problems, ease of access to locations such as parks and gyms for physical activity, inclement weather and competing priorities including work and social activities:

Because of my visual impairment I wouldn’t be able to see to operate the machine because it’s all digital, to see the numbers … So, I have to have someone to go with me … there have been occasions when I haven’t been able to go because the person is not able, he’s been otherwise preoccupied, had to do other things … I am sort of made a prisoner in my own area because of difficulties getting over that main road, because there’s no pedestrian button at the traffic lights or anything like that. (ID3, Female, aged 57, 19.7% CVD risk, 2nd IMD quintile)

Issues with the implementation of behaviour change support services deterred some participants from adopting risk‐reducing behaviours. One participant reported that she had not started going to the gym because she was told someone would be in touch about this, but she had not heard anything. Another turned up for an exercise class to be told it was full, and the alternative class suggested to her was held several miles away.

### 3.4 Perceived enablers to longer‐term change

Free gym access and exercise classes were viewed very positively and appeared to increase the likelihood of attendance.

It helped me to be put on a programme for the gym where I wouldn’t have to pay the full price. I could get to the gym for health reasons. A reduced price would help; financially … Because I’m registered disabled and am in receipt of the disability living allowance, or if you’re also on income support, I know I can access the gyms free. (ID3, Female, aged 57, 19.7% CVD risk, 2nd IMD quintile)

Some participants suggested it would be easier to maintain an exercise regime if there was some continuity in supportive follow‐up and feedback from a health or exercise professional or others in the same situation:

Once I’d lost a bit of weight that was lovely, as I say, the ‘group therapy’ thing because you went every week to be weighed and ‘oh you’ve lost another pound’. I think any group, when everyone does it, the group thing, is better than trying to do it on your own. (ID7, Female, aged 65, 16% CVD risk, 1st IMD quintile)

I’m working with a personal trainer and I’m starting to run and swim. He’s really smart and going at it the right way and I’m probably eating better. It takes my breath away (the cost) but I just decided I would spend it there to see if I can crank a few more years out of this body before it’s over! I have lost weight, I don’t look at the scales much. I just see how my clothes fit. (ID17, Male, aged 61, CVD risk >10%, 3rd IMD quintile)

People felt supported by close family members and friends joining in risk‐reducing behaviours, such as going for walks, runs or cycling together. Individuals appreciated receiving positive feedback on behaviour change and encouragement when maintaining behaviour change proved challenging, such as adhering to a healthier diet. Making changes together as a couple or a family appeared to support change:

I have tried with my wife to eat better things. We’re not on the junk food wagon, but trying to have more fish and we have plenty of fresh vegetables. We have slightly changed. I went on semi‐skimmed milk from the full fat variety, that sort of thing. (ID16, Male, aged 54, CVD risk >10%, unknown IMD quintile)

Older participants were sometimes motivated by wanting to be healthy as they went into retirement and older age:

So we’re trying to remove temptation … I regarded it as a useful prompt (the health check) because I had it in the back of my mind, that because we’ve both retired, it would benefit us (to change lifestyle). (ID10, Male, aged 66, 10.2% CVD risk, 1st IMD quintile)

Health check participants who were also health professionals themselves felt encouraged by the health check to make beneficial changes to their lifestyle, although others felt pressure to be a good role model to their patients, with one nurse explaining:

I basically have to set an example, because you’re constantly giving out the advice to your patients, to diet, because there’s a lot of obesity, diabetes and heart disease and all sorts of things, so you have to set an example. You can’t be telling people to do things if you yourself are not doing it. (ID14, Female, aged 56, CVD risk >10%, 2nd IMD quintile)

## 4 DISCUSSION

In this qualitative study, we aimed to develop an understanding of how higher‐risk patients who have undergone a health check are able to adopt and maintain behaviour change with risk‐reducing interventions. Overall, the participants interviewed welcomed the interventions provided. However, a minority were unaware they had received a health check. This might be due to their having an opportunistic health check, rather than responding to the health check invitation.

Few participants could recall their CVD risk score or appeared alarmed by the potential seriousness of having a score that exceeded a 10% risk. Lack of understanding about risk may have an impact on engagement with lifestyle change or decisions to take or continue with prescribed medication. Previous studies have reported that people found their CVD risk score confusing^10^ and that some individuals have unrealistically optimistic interpretations of their CVD risk.^24^ However, several participants could recall the meaning of the risk information. Moreover, in the current study, patients were interviewed six months post‐health check. Therefore, they may have understood their risk at the time of the health check but were not able to recall details of the feedback they have received at the time of the interview. Regardless of perceived risk, participants reported making lifestyle changes following the health check. This could suggest a weak effect of CVD risk communication on individuals’ intentions to change behaviour.^25^, ^26^ Welschen et al suggested that improvements in the perception of CVD risk did not influence patients’ attitudes and intentions to change their behaviour.^25^ The overall experience of attending the health check may have an impact on thinking about health and trigger behaviour change, regardless of CVD risk score.^27^, ^28^ This might be explained by the population who take up the offer of a health check, where people who respond to the health check invitation might be more motivated to adopt a healthier lifestyle.^29^

Some of those interviewed were aware of negative news reports of the adverse effects of statins, which may contribute to ambivalence to taking this medication. Negative media reports on statins have been associated with an increase in statin discontinuation rates.^30^ Concerns about the risks and side‐effects of statins and uncertainty regarding their benefits are key reasons for statin non‐adherence.^11^, ^31^ Other reasons for non‐adherence to long‐term medication use are requiring to build a routine, which takes time and work and heavily context dependent.^32^ Patients who were prescribed statin for CVD prevention often refer to taking statin as a need rather a choice.^33^ Patients reported feeling pressured by their doctors to start statin therapy, and others felt unaware of the possible side‐effects of statins and their pharmacological mechanism.^11^ Previous evidence suggested that patients viewed taking statin for CVD prevention as a means to mitigate against unhealthy lifestyles.^33^ This reflected this study participants wanting to try lifestyle changes before turning to medications. There is a need to address patients concerns and perceptions of statins to improve the health check programme’s overall outcomes. Addressing patients’ concerns could be done by discussing the reasons for prescribing statins and explaining possible side‐effects and ways to manage them. Higher‐risk patients in the current study rarely discussed their experience with antihypertensive medications. This could be due to the greater media coverage of statin use, lack of side‐effects associated with antihypertensive use or that hypertensive patients were prescribed less often than statins in the group we interviewed.

Although wanting to make lifestyle changes, some participants reported medical, physical and financial barriers to change, as has been found in previous studies.^10^ Patients reported difficulty changing behaviour because of stressful circumstances or work‐related constraints. Although previous studies suggested that older adults felt that changing their lifestyles at their age was unnecessary,^10^ older participants in the current study were motivated to change for a healthy retirement. Family history of CVD acted as a double‐edged sword in relation to behaviour change. Whilst for some a family history of CVD was a source of motivation to change, others felt that changing their behaviour will not alter their inevitable risk of CVD. This result echoes with previous findings that individuals with family history of heart diseases do not think that they can reduce their personal risk of developing heart diseases by changing their behaviour.^34^ Participants at high risk explained the importance of increased support and follow‐up to sustain behaviour change, either by family and friends or by health‐care professionals. Ismail and Atkin conducted a study to understand the experience of patients who went for the health check, regardless of their CVD risk level, and reported their perspectives on behaviour change.^35^ Participants in their study expressed the need for a proactive approach in advocating for lifestyle change by their health‐care providers.^35^ Social support appears to be a key factor not only for the initiation of behaviour change, but also for facilitating long‐term behaviour change maintenance.^36^ Previous evidence has suggested that health‐care professionals implementing the health check believed in the importance of providing continued support and follow‐up to encourage and maintain behaviour change.^37^ However, due to time constraints and workload pressures on primary care professionals this is not always feasible.^37^ Therefore, making referrals to external lifestyle interventions is believed to provide high‐risk patients with the support they need.

Lifestyle support services are in place to facilitate behaviour change and reduce barriers as part of the health check programme. However, these services are not always accessible or reliable.^10^, ^38^ Many of those we interviewed were referred to lifestyle change interventions including exercise and weight loss groups. However, there were often barriers to joining these interventions such as long waiting lists, distance from home and the timing of classes. The current study was conducted in the early stages of the programme implementation; therefore, referral schemes may have been not well developed then. A recent study has suggested similar difficulties to accessing lifestyle support services including long waiting lists, discontinuation of services due to budget cuts and services offered during working hours making it difficult to access by the working population.^37^ Improving the provision of these services is essential to provide patients with the needed continued support and follow‐up to adopt long‐term lifestyle changes.

Participants in this study were generally positive about the health check programme and motivated to improve their heath. However, this study highlights several areas where changes could be made to improve the programme’s outcomes. Raising awareness of CVD risk and lifestyle change opportunities is insufficient to reduce levels of CVD. The challenge lies in maintaining behaviour change in the long term. Even though patients are willing to change their behaviour to reduce their risk, behaviour change is heavily dependent on other factors, including socio‐economic circumstances and social support. Population‐level strategies that facilitate behaviour change through environmental changes may result in greater benefit,^39^ yet may need to be combined with interventions targeting individuals’ motivation and capabilities to maximize benefits.

### 4.1 Strengths and limitations

This study is amongst the first to examine adherence to behaviour change interventions and medications amongst higher‐risk individuals within the context of a health check programme. Interviewing higher‐risk individuals up to six months following the health check allowed for exploring barriers to long‐term change and medication initiation and/or adherence. This study included a sample of medium‐ to high‐risk men and women. The findings offer insights into factors associated with behaviour change in relation to participants’ social circumstances, such as their ability to access lifestyle change interventions and social influences.

The findings need to be assessed in relation to the design and setting of the study. Patients who do not attend the health check are also difficult to recruit in research, so we have not captured the views of people who may be less motivated to acknowledge and reduce their CVD risk. Our study did not include younger participants in their forties. People from this working‐age population might find it more difficult both to attend the health check^40^, ^41^ and to find the time to be interviewed. Although this was a study with a small sample in two socially deprived London boroughs and most participants were of white ethnicity, we interviewed a divers sample in terms of age, sex and socio‐economic status that may reflect the views of those eligible to be invited for a health check. However, it is possible that the views and experience of patients from other areas or ethnicities would differ. Potential participants might have been deterred from joining the study because they were invited by their GPs and because they had been identified via the health check as individuals who needed to change their behaviour and/or receive medication. This may have deterred some who felt some stigma attached to their lifestyle choices. No other means of recruitment was available, however, and we did get a low response rate, although this level of recruitment is fairly standard in this area of South East London. We were unable to recruit further participants during the timeframe of the study. If we had been able to recruit for longer, we may have ended up with a more diverse sample and captured a wider range of views. It is possible that data collection and interpretation were influenced by the research team’s background in health psychology and public health. Analysts from different disciplines may have developed an alternative analytic framework. Finally, face‐to‐face interviews may be preferable as the interviewer can be aware of non‐verbal cues. However, face‐to‐face interviews to discuss behaviour change can produce social desirability bias.^42^ The participants might have given responses that they perceive as socially acceptable. In our study, the quality of the telephone interviews was good and it may have been easier for some participants to discuss issues, such as weight, without concerns about being judged by the interviewer.

## 5 CONCLUSIONS

This study suggests that people are generally motivated and willing to change their behaviour following the health check, regardless of their understanding of their personal risk of a cardiovascular event. Easy access to services to support behaviour change, however, is variable. Concerns over the side‐effects of medications need to be addressed in order to improve adherence. Strategies are required to facilitate long‐term behaviour change maintenance, possibly through greater provision and support of lifestyle change services.

## CONFLICT OF INTEREST

The authors declare no conflict of interest regarding this research.

## Data Availability

The data are not publicly available due to privacy and ethical restrictions.
