# Supplementary material for: Engagement with advice to reduce cardiovascular risk following a health check programme: A qualitative study
Source: Health Expect. 2019 Oct 23;23(1):193–201. doi: 10.1111/hex.12991 (PMC6978858; doi:10.1111/hex.12991)
Supplement: Supplementary file 1 [file HEX-23-193-s001.docx]

Supplementary material 1: Interview schedule.

Exploring patients' experiences of NHS Health Checks (3)

Interview Schedule

Introduction

Thank you for agreeing to take part in our study. We are looking at people’s experience of having an NHS Health Check. We are interested in what people in South London think about the NHS Health Check and their reactions to the information they are given. I just want to ask you a few questions about your thoughts – please feel free to answer as honestly as possible. There are no right or wrong answers. All information will be kept completely confidential. Taking part in the research will not influence the care you receive from the NHS. If you would prefer not to answer any of the questions that is fine and we can stop the interview at any point if you do not wish to continue. Are there any questions you would like to ask me before we start? (Turn on recorder)

- When did you receive your health check? (Approximately)
- Did you receive a letter in the post inviting you to have a health check? (Or was it done when you were attending the GP for another reason? Or did you respond to an advert e.g. in the pharmacy?)
- Who conducted (did) your health check (doctor, nurse, pharmacist, other)?

Semi-Structured Interview Schedule

***The health check***

- What were your expectations about the Health Check? What did you think would happen at the appointment?
- What were you told when you had your health check*? What did they tell you e.g. about your risk of developing cardiovascular disease (heart attack or stroke)?*
  - *What did you think of that?*
  - *(if gives result in numbers) What size of risk would you say that was?*
- How did you feel when you were told your test result?
- How was the information given to you? *(e.g. with computer images, leaflet etc)*
- What, if anything, did they suggest you do as a result of your test? What were you asked to consider doing? *(e.g. medication, lifestyle change)*

***Medication (if medication was advised)***

- You mentioned it was suggested you take some medication, how did you feel about that? *Did you feel it made sense (to take medication)?*
- How important did you think it was for you to take the medication? (tapping perceived necessity)
- Did you have any concerns about taking medication? What were they?
- Did you talk to anyone about your decision to take the medication? What did they think?
- Did you decide to take the medication? How have you been getting on with it? *Or: Why did you decide not to take it?*
- Any practical problems with taking the medication? *e.g. obtaining repeat prescriptions, remembering to take it?*
- How did you try and overcome these problems, if at all?
- Is there anything that would have made it easier?
- What did/do you hope taking the medication will achieve?

***Lifestyle change (if this was advised)***

- You mentioned it was suggested you might take more exercise/stop smoking/change diet...
- What did you think of that?
- How confident were you about changing your behaviour?
- Did the person doing the check suggest where and how you might do this? ***[Go through each suggested lifestyle change]***  *E.g. via a lifestyle “hub”, local gym or leisure centre?*
- *Do you think that suggestion was right for someone like you?*
- Did anyone else get in touch with you after the check to discuss lifestyle change? *E.g. health trainer or practice nurse by phone, letter, email*
- Did you have any concerns about taking up these suggestions? What were they?
- Did you discuss it with anyone? *(e.g. partner, work colleague, friend)?*
- Did you decide to take this up? If not, what stopped you? If yes, how have you been getting on with it?
- What did you hope it (the lifestyle change) would achieve?
- Overall, would you recommend this activity/class to someone else?
- What problems, if any, have you experienced in making this change in your lifestyle?
- Did you try to do anything about these problems? (if so) What did you try? How did that go?
- Is there anything that would have made it easier?
- Were there things (other priorities) going on in your life that got in the way of [doing behaviour]
- Do you think you’ll be able to keep going with [behaviour] in the long term? Why is that?
- ***Access/environmental factors (if not elicited by above questions)***
- How do/did you get there *(to exercise class, smoking cessation, weight loss meeting etc)?*
- How convenient was/is this? *(e.g. transport difficulties; class times; how it fits in with work/ family; health/mobility problems)*
- Do financial factors make a difference to whether or not you go to behaviour change activities, or whether or not you take your medication? (E.g. time to collect prescription, cost of prescription)
- Would you like to add anything in relation to what we’ve been talking about?

**.......................................................................................................................................................**

Thank you

Confidentiality

Any questions about the research

Complete Demographics
